# Supplementary material for: Forecasting weekly dengue incidence in Sri Lanka: Modified Autoregressive Integrated Moving Average modeling approach
Source: PLoS One. 2024 Mar 8;19(3):e0299953. doi: 10.1371/journal.pone.0299953 (PMC10923413; doi:10.1371/journal.pone.0299953)
Supplement: Attachment — Submitted filename: Response to the Reviewers.docx [file pone.0299953.s002.docx]

Dear Mohamed R. Abonazel,

Academic Editor

PLOS ONE

We would like to express our profound appreciation to you for the valuable comments and suggestions made on our manuscript which were very helpful in revising and improving it. Please note that revised manuscript with track changes has been attached under 'Other’ category.

**Point by point response to editor**

| **Comment from Editor** | **Response form Authors** |
| --- | --- |
| I also noticed that the submission does not meet our publication criterion #5 (http://journals.plos.org/plosone/s/criteria-for-publication#loc-5) which states that articles must be presented in an intelligible fashion and written in standard English. | The manuscript has been edited to correct mistakes in punctuation. Additionally, rephrasing has been done to ensure intelligible presentation, with mild revisions made to the substance. Emphasis has been placed on highlighting the novelty of the research, and contextual revisions have been made accordingly. These efforts have improved the overall quality, coherence, and readability of the manuscript. Please refer to the attached manuscript, which is categorized under 'Other'. |
