# Supplementary material for: Forecasting weekly dengue incidence in Sri Lanka: Modified Autoregressive Integrated Moving Average modeling approach
Source: PLoS One. 2024 Mar 8;19(3):e0299953. doi: 10.1371/journal.pone.0299953 (PMC10923413; doi:10.1371/journal.pone.0299953)
Supplement: Attachment — Submitted filename: Response to Reviewers.docx [file pone.0299953.s003.docx]

**Point by point response to editor and reviewers.**

Dear editor and the reviewers,

We would like to express our profound appreciation to the editor for giving us the opportunity to revise and resubmit the paper. We are also grateful to the reviewers for the constructive comments and suggestions made on our manuscript which we found very helpful in revising and improving our paper. Given below is a detailed description of how we have addressed each comment in the revised version of the paper.

| **Comments from the Editor** | **Comments of the authors** |  |
| --- | --- | --- |
| The authors are requested to make appropriate modifications to this manuscript as suggested by the reviewers | We have addressed all points raised by the editor and both reviewers and are given below. |  |
| 1. The manuscript must describe a technically sound piece of scientific research with data that supports the conclusions. Experiments must have been conducted rigorously, with appropriate controls, replication, and sample sizes. The conclusions must be drawn appropriately based on the data presented.  Reviewer #1: Partly  Reviewer #2: Partly | Well noted your comment with thanks.  In our study, experimental procedures were not conducted as our research relied solely on secondary data analysis. The dataset used for model training comprised weekly dengue count data spanning from 01 January 2015 to 30 August 2020, while data from September 2020 to December 2020 were held out for model validation. We meticulously modified the conclusion section to accurately reflect the findings presented in the study. Additionally, to enhance the conclusion's comprehensiveness, we incorporated six additional lines at the outset of the conclusion, further reinforcing the significance and implications of our research. |  |
| 2. Has the statistical analysis been performed appropriately and rigorously?  Reviewer #1: Yes  Reviewer #2: Yes | Well noted your comment with thanks. |  |
| 3. Have the authors made all data underlying the findings in their manuscript fully available?  Reviewer #1: Yes  Reviewer #2: Yes | Duly noted! Thank you! Response well considered |  |
| 4. Is the manuscript presented in an intelligible fashion and written in standard English?  PLOS ONE does not copyedit accepted manuscripts, so the language in submitted articles must be clear, correct, and unambiguous. Any typographical or grammatical errors should be corrected at revision, so please note any specific errors here.  Reviewer #1: Yes  Reviewer #2: No | We sincerely appreciate the valuable suggestion provided by the reviewers. To ensure the utmost quality and clarity of the revised manuscript, we have taken significant measures to guarantee that it is written in standard English, free from any typographical or grammatical errors.  In pursuit of this objective, we sought professional proofreading services from a Senior Lecturer in English within our esteemed Faculty of Humanities and Sciences at SLIIT University. Their expertise and meticulous review have further enhanced the manuscript's language and readability, reinforcing our commitment to maintaining the highest standards of scholarly communication.  We are grateful for the opportunity to improve our work through these meticulous revisions, and we eagerly anticipate the possibility of sharing this enhanced version with the scientific community through the esteemed pages of this journal. |  |
| **REVIEWER #1** | | |
| **Comments from the Reviewer 1** | **Comments of the authors** |  |
| 1. The modeling and the method of doing and reporting it are approved**,** but it is necessary to report and prepare the article based on the guidelines of the journal. | We sincerely value your valuable comment, and in response, we diligently prepared the revised manuscript in full compliance with the journal's guidelines. |  |
| 2. The Literature Review section should be removed, and it is better to integrate it in the introduction as a summary | We sincerely appreciate the invaluable feedback provided by the reviewer. Recognising the merit of their suggestion, we have proceeded to remove the Literature Review section and skilfully integrated its essential aspects into the Introduction. Furthermore, to enrich the scholarly foundation of our work, we have thoughtfully incorporated additional references as advised by Reviewer 2.  This can be found from the revised version, lines from 83-111, 115-131, 139-141 and 152-153. |  |
| 3. Figure 6 and 7 should be merged. | We are immensely grateful for the valuable comment provided by the esteemed reviewer. We acknowledge and agree with their suggestion to merge Figure 6 and 7. However, after careful consideration, we found that merging the figures compromised clarity due to the inclusion of actuals, predicted, and ± 2SE.  Our original intention was to present the actual and predicted values separately for both the training set and the independent validation set. As a result, we have decided to remove Figure 6 and incorporate the relevant information into Figure 7 to maintain a clear and concise representation of the data.  Furthermore, we have taken into account the constructive input from Reviewer 2 and have now included the Mean Absolute Percentage Error (MAPE) for both training and validation data sets as suggested. This addition strengthens the reporting of the model's performance and ensures the completeness of our analysis.  Once again, we express our appreciation for the reviewer's valuable feedback, which has undoubtedly improved the presentation and accuracy of our research. We are confident that these revisions will enhance the overall quality of the paper and contribute to its impact within the scientific community. |  |
| 4. What is the reason for the difference between the actual and predicted values in Figure 8? | We extend our sincere gratitude to the esteemed reviewer for their insightful query regarding the discrepancy between the actual and predicted values in Figure 8. We appreciate the opportunity to address this concern in a more professional manner.  It is essential to recognise that achieving a perfect model, where the actual and predicted values are exactly the same, is an ideal scenario that is rarely attainable in practical applications. In real-world situations, the presence of errors is inevitable due to various factors that affect the model's performance.  As we have duly emphasised in the manuscript, the existence of a difference between actual and predicted values is inherent in predictive modeling. This disparity is commonly known as the prediction error, and its presence is a natural consequence of the model's attempt to capture complex relationships and patterns within the data.  Our intention was never to imply that there should be no difference between actual and predicted values. On the contrary, the focus of our analysis was to ensure that these prediction errors do not exhibit any discernible systematic pattern, as such patterns would indicate potential issues with the model's performance and validity.  In the manuscript, we have presented comprehensive evidence that demonstrates the random nature of the prediction errors. Our careful evaluation of the residuals confirms that they exhibit no systematic patterns, supporting the robustness and reliability of our modeling approach.  We appreciate the reviewer's attention to this critical aspect of our research, as addressing the presence and randomness of prediction errors is essential in the context of predictive modeling. By providing this clarification, we aim to reinforce the accuracy and validity of our findings, and we hope it further enhances the overall quality and impact of our paper. In the revised version, you can find this information spanning from line 406 to line 445 (406-412, 416-422 and 432-445).  Once again, we express our gratitude to the reviewer for their thoughtful feedback, which has undoubtedly contributed to the refinement and advancement of our work. |  |
| 5. Goodness-of-fit of the final model such as AIC, BIC, normality of the residuals, mean absolute percentage error (MAPE) index, etc. should be reported. | Thank you for highlighting the importance of reporting the goodness-of-fit measures for the final model, including AIC, BIC, normality of residuals, and the Mean Absolute Percentage Error (MAPE) index.  We are pleased to inform the reviewer that we have already addressed their suggestions and incorporated all the mentioned indicators in the revised manuscript. Specifically, AIC and SBIC have been included in Table 2, providing a comprehensive view of the model's fit and performance.  It is worth noting that BIC and SBIC are equivalent in some contexts, as BIC is occasionally referred to as the Schwarz Bayesian Information Criterion (SBIC). Therefore, in our analysis, both terminologies refer to the same information criterion and have been appropriately presented in the relevant sections of the paper.  By integrating these essential measures into our study, we have ensured a thorough and rigorous evaluation of the model's goodness-of-fit and predictive accuracy. These metrics contribute significantly to the validity and reliability of our findings and conclusions. The revised text contains the relevant details, accessible from line 387 to line 389.  Once again, we express our gratitude to the reviewer for their invaluable input, which has positively influenced the completeness and robustness of our research. |  |
| 6. The article is long; it should be written more concisely | We appreciate the reviewer's feedback, and we agree with their suggestion to write more concisely. In response, we have diligently reduced the length of the revised manuscript while maintaining the core contributions. This has led to a more impactful and accessible presentation of our research findings. Thank you for the valuable opportunity to improve our work. |  |
| **REVIEWER #2** | | |
| **Comments from the Reviewer 2** | **Comments of the authors** |  |
| 1. The authors have focused on accurately forecasting dengue infections by utilizing historical data on infected cases. To achieve this, they have employed statistical models presented in time series analysis. Based on the data collected in the Colombo district, Sri Lanka, the manuscript concludes that ARIMA models are suitable for dengue forecasting.  The current title of the manuscript does not clearly reflect the objective/s. The phrase "Changing face of dengue" in the title implies a different meaning than what the authors intended. Therefore, the title should be changed to align appropriately with the content. However, the abstract has properly summarized the content and the objectives, and the conclusion of the study. | We sincerely appreciate the valuable feedback provided by the esteemed reviewer concerning the title of our manuscript. We agree that the previous title did not accurately reflect the objectives and content of the study.  In response to this insightful suggestion, we have taken appropriate action and revised the title to align more appropriately with the research conducted. The new title now reads, "Forecasting Weekly Dengue Incidence in Sri Lanka: A Modified Autoregressive Integrated Moving Average Modeling Approach."  Furthermore, we have duly noted that no comments were received regarding the abstract from both reviewers, and thus, we have retained the original abstract as it effectively summarises the content and objectives of our research.  Thank you for your careful evaluation of our work, and we eagerly await the possibility of sharing this improved version of our manuscript. |  |
| 2. The graphics included in the manuscript are often in the form of pictures instead of vector images, which has led to a decrease in image clarity and quality. Additionally, software-generated outputs have been directly presented as tables, as evident in Table 3. This approach results in the overcrowding of information within a single table, leading to poor clarity and readability. | We sincerely appreciate the valuable feedback provided by the esteemed reviewer regarding the quality of Tables and Figures in our manuscript. Their constructive comments have been instrumental in enhancing the visual presentation of our research.  In response to their insightful observations, we have taken the following actions to improve the clarity and readability of our Tables and Figures:  Figures:   1) Figure 1: We have made significant improvements to Figure 1 to ensure enhanced clarity and quality. 2) Figure 2: While Figure 2 remains unchanged, we have added a footnote to provide additional context and clarification. 3) Figure 3: To avoid direct reliance on software-generated outputs, we have recreated Figure 3 using Excel graphs, thereby improving image quality without compromising on the accuracy of the information. 4) Figure 4 and Figure 5: We have enhanced both Figure 4 and Figure 5 by removing software-generated lines, resulting in clearer visuals.   Figures that are no longer relevant or essential for the manuscript have been removed as per the reviewer's suggestion. Specifically, we have removed Figure 6 and made necessary improvements to Figures 7 and 8 to ensure consistency and coherence in the overall presentation.  Tables:   1) Table 1: To enhance the readability, we have reduced the number of rows by adjusting the width of column 1. 2) Table 2: We have retained Table 2 without any changes, as it effectively presents the necessary information. 3) Table 3: We have retyped Table 3 to eliminate the presentation of software-generated outputs directly. This has improved the table's organisation and readability. We have also excluded certain software-generated outputs, such as R^2^ and AdjR^2^, which are not crucial for ARIMA models.   These meticulous revisions have significantly improved the visual appeal and overall quality of the manuscript, aligning it with the high standards set by the journal.  We are sincerely grateful for the reviewer's attention to detail and their valuable input, which has greatly contributed to the refinement of our work. |  |
| 3. In the literature review, various models used in dengue forecasting have been discussed. However, there is an insufficient presentation regarding the application of ARIMA/AR models in dengue forecasting, despite them being the underlying models used in the current research. The keywords: ARIMA + "Dengue forecasting" resulted in numerous related articles | We express our sincere gratitude to the esteemed reviewer for providing valuable feedback on the literature review section of our manuscript. Your observation regarding the application of ARIMA/AR models in dengue forecasting is duly noted.  In response to your insightful comments, we have taken decisive action to address this concern. As suggested by Reviewer 1, we have removed the dedicated Literature Review section and instead, we have incorporated a concise summary of relevant literature on dengue forecasting, including the application of ARIMA/AR models, within the Introduction.  While we believe that the literature review covered a substantial number of relevant papers, we acknowledge the need to further emphasise the application of ARIMA/AR models in our research. To this end, we have included two additional references (17, 12) that specifically highlight the utilisation of ARMA models for forecasting dengue and identifying factors influencing its occurrence.  By incorporating these new references, we have strengthened the foundation of our study and ensured that the essential contributions of ARIMA/AR models in dengue forecasting are appropriately represented.  Once again, we sincerely appreciate your insightful comments, which have enabled us to refine the manuscript and provide a more comprehensive overview of the relevant literature in the field of dengue forecasting. |  |
| 4. In dengue cases forecasting, a weekly resolution of the data is considered suitable for time series analysis. Therefore, the study utilizes appropriate secondary data for its analysis. Since the ARIMA and AR models are standard models, a detailed description is not necessary. The authors can provide a concise introduction to these models in the introduction section. Generally, a comprehensive explanation of fundamental time series techniques is not expected in an article of this nature. Thus, it is strongly recommended to condense the details and cite relevant content for further clarification. Additionally, the authors have used notations without properly introducing them in advance (refer to lines 173, 174). There are many typing mistakes visible in the manuscript; refer to lines 61 and 98. Further, sentence structure has to be improved. | We sincerely appreciate the invaluable feedback provided by the esteemed reviewer regarding the literature review and technical aspects of our manuscript. Your observations regarding the presentation of ARIMA/AR models in dengue forecasting are well-received, and we acknowledge the need for improvement.  Regarding the weekly resolution of data for time series analysis in dengue cases forecasting, we have taken this into account and utilised appropriate secondary data for our analysis. We recognise that ARIMA and AR models are well-established and widely used in time series analysis, and therefore, a detailed description of these models is not essential in this context. Instead, we will provide a concise introduction to these models in the Introduction section, highlighting their relevance to our research.  We understand that comprehensive explanations of fundamental time series techniques may not be expected in an article of this nature. To address this, we will condense the technical details and cite relevant content for further clarification, ensuring that the manuscript remains focused and concise.  Additionally, we are grateful for bringing to our attention the use of notations without proper introduction (lines 202, 203), as well as the presence of typing mistakes (lines 73 and 113). We will carefully review the manuscript to rectify these errors and ensure that all notations and terms are appropriately introduced for clarity.  We sincerely appreciate the reviewer's attention to sentence structure and will make the necessary improvements to enhance the overall readability and flow of the manuscript.  In conclusion, we value the thorough evaluation and constructive feedback provided by the reviewer. Your input will undoubtedly contribute to the refinement and enhancement of our research. We are committed to delivering a professionally written and error-free manuscript that effectively communicates our findings to the scientific community. Thank you for the opportunity to improve our work, and we look forward to the possibility of sharing this improved version through the esteemed pages of this journal. |  |
| 5. As mentioned previously, the existing literature discusses the appropriateness of using ARIMA models for dengue forecasting. However, the suitability of applying ARIMA models to the specific dataset may not have been thoroughly investigated. Given that dengue dynamics are influenced by the interactions between vectors and humans, it is reasonable to assume that ARIMA models could be applicable to the underlying dataset. To truly contribute to the existing literature and establish novelty in the research, it is important for the authors to present significant results that demonstrate the adequacy of the ARIMA models rather than solely focusing on model diagnostic results. One approach to achieve this is by analyzing the robustness and stability of the ARIMA model(s) in forecasting dengue cases across different contexts. For example, examining the accuracy of forecasting extreme events such as the peak of dengue incidents in 2017. Neglecting to address these aspects raises doubts about the novelty of the present study. The findings should go beyond simply fitting a time series model to a given dataset. The results should provide meaningful insights into the forecasting capabilities of ARIMA models for dengue, as ARIMA is not new in dengue case forecasting | We sincerely appreciate the thoughtful and constructive feedback provided by the esteemed reviewer regarding the suitability and novelty of our ARIMA model for dengue forecasting. We acknowledge the importance of establishing the adequacy and robustness of the ARIMA model in the specific dataset to contribute meaningfully to the existing literature.  To address these aspects, we have made the following enhancements in the manuscript:   1) Novelty Explanation: We have further elaborated on the novelty of our ARIMA model in the Research and Development section and in the Conclusion. Specifically, we emphasise that our model distinguishes itself from traditional ARIMA models by incorporating an AR(16) parameter, which enables improved forecasting accuracy. This can be found from the revised version line 543 to line 545 and from 550 to 558. 2) Robustness and Stability: We have thoroughly discussed the robustness and stability of the ARIMA process. To demonstrate the accuracy of our forecasts, we have included the Mean Absolute Percentage Error (MAPE) as suggested by the reviewer. Additionally, we have analysed the roots of the ARIMA models to ensure stability. The required details lie within the textual segment encompassing lines 397 to 399. 3) Examining Extreme Events: To showcase the forecasting capabilities of our ARIMA model, we have analysed extreme events, such as the peak of dengue incidents in 2017. This examination provides meaningful insights into the model's performance and its ability to handle varying contexts. For the required details, kindly examine the content located between lines 432 and 446.   By addressing these aspects, we have ensured that our findings go beyond simply fitting a time series model to a given dataset. Instead, we now provide significant results that highlight the adequacy, robustness, and forecasting capabilities of our ARIMA model for dengue.  We genuinely appreciate the reviewer's attention to detail, which has helped us strengthen the manuscript's content and contribute more effectively to the scientific understanding of dengue forecasting. We remain committed to delivering a professionally written and rigorously analysed manuscript that advances the knowledge in this field.  Thank you for the opportunity to improve our work, and we eagerly anticipate the possibility of sharing this enhanced version through the esteemed pages of this journal. |  |
| 6. Based on the issues that have been highlighted regarding the novelty, contribution, clarity, and structure of the manuscript, it seems that significant revisions and improvements are necessary for it to be considered suitable for publication. It's important to address these concerns and make the necessary enhancements to ensure the manuscript meets the standards expected in the field. | We sincerely appreciate the thorough evaluation and constructive feedback provided by the esteemed reviewers. Their valuable comments have been instrumental in identifying areas for improvement in our revised manuscript.  In response to the issues raised by both reviewers, we have undertaken significant revisions to address the concerns and enhance the quality of the manuscript. Our aim is to ensure that it meets the high standards expected in the field of dengue forecasting.  We have carefully considered each comment and made the necessary enhancements to the novelty, contribution, clarity, and overall structure of the paper. By incorporating these improvements, we are confident that the revised manuscript now presents a more impactful and rigorous contribution to the scientific literature.  We thank the reviewers for their thoughtful evaluation, which has guided us in refining our work. We remain committed to delivering a professionally written and well-structured manuscript that effectively communicates our research findings to the scientific community.  Once again, we express our gratitude for the valuable feedback, and we eagerly await the opportunity to share this improved version of our research through the esteemed pages of this journal. |  |
