# Supplementary material for: Forecasting weekly dengue incidence in Sri Lanka: Modified Autoregressive Integrated Moving Average modeling approach
Source: PLoS One. 2024 Mar 8;19(3):e0299953. doi: 10.1371/journal.pone.0299953 (PMC10923413; doi:10.1371/journal.pone.0299953)
Supplement: Attachment — Submitted filename: Response to Reviewers.docx [file pone.0299953.s004.docx]

**Point by point response to editor and reviewers.**

Dear editor and the reviewers,

We would like to express our profound appreciation to the editor for giving us the opportunity to revise and resubmit the paper. We are also grateful to the reviewers for the constructive comments and suggestions made on our manuscript which we found very helpful in revising and improving our paper. Given below is a detailed description of how we have addressed each comment in the revised version of the paper.

| **Comments from the Editor** | **Comments of the authors** |
| --- | --- |
| 1. To ensure that the estimated model fits the data, the authors should perform the test of normality of the residuals as a required diagnostic test for the ARIMA model. | We wish to extend our profound gratitude to the reviewer for their invaluable insights pertaining to the diagnostic assessments of the ARIMA model. It has come to our attention that this critical dimension had been inadvertently omitted from our preceding manuscript. We hold in high regard the reviewer's diligence in identifying this omission, and we are sincerely appreciative of their efforts in doing so.  In response to this constructive feedback, we undertook a comprehensive evaluation of the residuals, focusing on their adherence to a normal distribution. We are pleased to report that this scrutiny yielded affirmative results, affirming the conformity of the residuals to a normal distribution. This affirmation was substantiated through a meticulous analysis of the residuals' histogram, thereby bolstering the credibility of our model.  To further buttress the empirical underpinnings of the normality of residuals in our ARIMA model, we have thoughtfully integrated the following scholarly reference into our revised manuscript:  Awwad FA, Mohamoud MA, Abonazel MR (2021) 'Estimating COVID-19 cases in Makkah region of Saudi Arabia: Space-time ARIMA modeling.' PLoS ONE 16(4): e0250149. <https://doi.org/10.1371/journal.pone.0250149>  This citation has been thoughtfully incorporated into the closing paragraph of page 26, serving to augment the empirical foundation of our methodological approach.  Additionally, we have included the information about the normality of residuals in the abstract on line 41.  Once more, we wish to convey our heartfelt appreciation to the reviewer for the constructive feedback, which has undeniably contributed to the enhanced rigor and quality of our research endeavour. |
| There are some grammatical and spelling errors in the paper, the authors need to carefully check the full text | In our unwavering commitment to upholding the pinnacle of quality and lucidity in our revised manuscript, we have undertaken substantial steps to guarantee its adherence to standard English, devoid of any typographical or grammatical imperfections.  In the pursuit of this aspiration, we engaged the services of a distinguished Senior Lecturer in English, a member of the esteemed Faculty of Humanities and Sciences at SLIIT University. Their consummate expertise and fastidious review process have yielded marked enhancements in the linguistic precision and overall readability of the manuscript, thereby underlining our steadfast dedication to upholding the loftiest benchmarks in scholarly discourse. As an example, we have modified the abstract to improve its style without changing the content. |
|  |  |
| **REVIEWER #3** | |
| **Comments from the Reviewer 3** | **Comments of the authors** |
| (1) Establish a common-sense baseline for comparison in Figure 1. For example how accurate would a model be that predicted the next week of cases would the same as the last. Would this outperform the ARIMA (2,1,0) model. If so what other advantages would the ARIMA (2,1,0) model hold. | We wish to express our gratitude for your invaluable suggestion. We fully acknowledge the significance of establishing a pragmatic baseline for comparison as depicted in Figure 1. Notably, a predominant portion of the dataset consistently exhibited values below 1000. However, it is worthy of note that a remarkable surge in cases occurred from the 2^nd^ week of June to the 1^st^ week of September, with reported figures ranging between 1032 and 1958. From a statistical standpoint, these data points were identified as outliers through conventional methodologies such as the interquartile range technique and Z-score analysis.  Our decision not to eliminate these outliers stems from the realisation that they represent genuine observations and may impart meaningful insights into our modelling endeavours. Consequently, to accommodate the variability introduced by these high-value data points, we introduced an additional AR(16) term to the ARIMA(2,1,0) model.  In response to your valuable suggestion, we have thoughtfully incorporated the subsequent statement within the manuscript to enhance its clarity:  " Fig 1 highlights a period of significantly elevated dengue cases, spanning from the second week of June to the first week of September. It is worth noting that since the country's initial dengue outbreak in 1965 – 1966, the highest incidence was reported in 2017, with a staggering 186,101 cases. Markedly, more than 25% of the country's dengue cases were concentrated within the Colombo district during this period.  These data points clearly exhibit outlier characteristics when assessed using both the interquartile range method (Q1-1.5IQR, Q3+1.5IQR) and a common heuristic criterion (z-score exceeding 3 or falling below -3). Nevertheless, it is imperative to underscore that, during the formulation of our ARIMA model, we refrained from excluding these data points on the grounds that they represent genuine and observed values integral to the current analysis. Furthermore, as portrayed in Fig 1, the time series plot underscores the non-stationary nature of the original observed series.  This addition has been meticulously placed in the line 315. We deeply appreciate the reviewer's perspicacious input, as it has undeniably contributed to the elucidation and robustness of our study. |
| (2) The authors identify three ARIMA candidate models [(0,1,2), (2,1,0), and (2,1,2)] for consideration. Two are deemed invalid because they do not match theoretical assumptions. However, readers will be curious if they would outperform the ARIMA (2,1,0) model in the evaluation. Including this data in the evaluation and discussing it would improve the paper. | We extend our sincere appreciation for your astute comments aimed at enhancing the caliber of our paper, and we have diligently taken steps to address them.  Our initial approach adhered to the conventional model selection process, which led to the identification of three potential models: ARIMA (0,1,2), ARIMA (2,1,0), and ARIMA (2,1,2). It is evident that ARIMA (2,1,2) can be confidently disregarded, given the insignificance of all its parameters. However, as you rightfully emphasized, the comparison between ARIMA (2,1,0) and ARIMA (0,1,2) for forecasting purposes could have been explored, as both models feature significant parameters.  Our preference for modeling with ARIMA (2,1,0) stems from its ability to effectively integrate the influence of past values directly into the model, an approach better suited to the characteristics of our dataset. Furthermore, it is noteworthy that ARIMA (0,1,2) exhibits significantly higher σ^2^ volatility in comparison to ARIMA (2,1,0). Consequently, our choice to evaluate these two candidates hinged on factors beyond just forecasting outcomes, considering various other statistical indicators.  In alignment with your valuable suggestion, we have thoughtfully incorporated the ensuing statements to augment the content of the paper:  "The outcomes presented in Table 2 reveal that all parameters within the ARIMA (2,1,2) model lack statistical significance, rendering it an unsuitable choice for further consideration. Conversely, the results in Table 2 demonstrate that all parameters within the ARIMA (2,1,0) and ARIMA (0,1,2) models are statistically significant, thus establishing both as potential candidates for forecasting. However, given that conspicuous outliers were not excluded from the original series, from a modelling perspective, ARIMA (2,1,0) emerges as the more favourable candidate. This is attributed to its capacity to directly capture the influence of past values, distinguishing it as a more robust choice compared to ARIMA (0,1,2)…."  This addition has been meticulously inserted in the line 383 to 390, enhancing the clarity and comprehensiveness of our manuscript. |
| (3) Furthermore, showing that the ARIMA(2,1,0) outperforms the ARIMA alternatives and the common sense baseline by a statistically significant margin would strengthen the contribution of the paper a great deal. This would demonstrate that the ARIMA(2,1,0) approach is materially more accurate than the alternatives and that improved accuracy will generalize | We wish to express our gratitude for your invaluable input. We acknowledge the critical importance of substantiating the superiority of the ARIMA (2,1,0) model over alternative models and the pragmatic baseline with a statistically significant margin. We are fully aware that achieving this would substantially enhance the paper and underscore the practical efficacy of our approach. |
| (4) The authors use one training/evaluation split for the data. The results are a little uninspiring as the forecast data does match the actual data in terms of data points or shapes. Were other training/evaluation splits considered. If so, was the performance ever better than what was shown. The paper would benefit from a larger presentation of different splits (i.e. more training data, less training data, conclusions about accuracy w.r.t. training data). | We greatly appreciate your considerate feedback. Your point regarding the examination of different training and evaluation data splits is certainly valid, and we recognise the potential impact on the model's performance.  While it is true that adjusting the composition of the training data can result in varying models, we fully comprehend the necessity for a more comprehensive analysis in this regard. We are committed to exploring the effects of altering the size of the training data and assessing how it influences the performance of the ARIMA (2,1,0) model. This endeavour will enable us to offer a more holistic perspective on the model's robustness across different scenarios.  Regarding the disparities between forecasted values and actual data in the independent dataset, we have already addressed this issue in lines 489 to 491. However, we intend to expound upon this matter further and provide an in-depth discussion concerning the model's forecasting performance in relation to different training and evaluation data splits, thus ensuring a more comprehensive presentation of our research findings. |
| In addition, the paper would benefit from a discussion of limitations. As it currently stands it appears as if all projections are made at the same geographic level. Identifying that research (see paper below) has shown that effective time series projections of viruses can depend on the geographic-level of evaluation (i.e country vs. city vs. county vs. neighborhood). Identifying exactly which of these units is used in the paper (i.e. country) and explicitly stating that changing this context might change the results of the analysis and the conclusions would help readers understand the context in which the results will generalize.  Lynch, Christopher J., et al. "Short-range forecasting of COVID-19 during early onset at county, health district, and state geographic levels using seven methods: comparative forecasting study." Journal of medical Internet research 23.3 (2021): e24925 | We express our gratitude for the thoughtful suggestion provided by the reviewer concerning the imperative need to address limitations, particularly in the context of the geographic level of evaluation. In the current iteration of our work, it is indeed the case that our projections are exclusively conducted at the national level.  We fully recognise the significance of the geographic level at which virus time series projections are formulated, as it can exert a considerable influence on the outcomes and the ability to extrapolate findings to broader contexts. In light of this, we will augment the paper's clarity by explicitly stating that our analysis is confined to the national level (country). We will also underscore the potential impact of altering this spatial context, such as conducting evaluations at a more granular scale, encompassing cities, counties, or neighbourhoods. This consideration was emphasised to convey that such adjustments may yield divergent results and conclusions.  Furthermore, we have made a conscientious effort to reference pertinent research, including the work by Lynch et al. (2021), in order to underscore the critical importance of taking into account the geographic level in the domain of virus forecasting.  A new section titled "Limitation of the study" has been included at line 631 on page 39. The inclusion of these discussions pertaining to limitations will serve to enhance the paper's transparency and provide readers with a more comprehensive understanding of the contextual boundaries within which our research findings can be applied and extended. |
| (5) The numbers in Q-STAT column of Figures 2 and 4 in the paper are difficult to compare to one another because the numbers are not right justified. As a result, the significant digits of the numbers are not aligned on top of one another (i.e. 100ths, 10ths, etc.) Right justifying the numbers in the table will improve readability and enable readers to compare results between rows.  For the data availability, the text points at Supporting Information 1. However, all that is included is the case data. Including the raw data for the analyses done to chose the model as well as the source code used to build the models, tables and plots in the paper would improve the availability of data for the paper. | We appreciate your comment regarding the arrangement of numbers within the Q-STAT column in Figures 2 and 4. Your recommendation to right-justify the numbers in order to enhance readability and facilitate comparison was well-received. Despite the fact that the output was generated by computer, we undertook a comprehensive review of formatting possibilities and examined the feasibility of aligning the numbers to achieve greater consistency in displaying significant digits. Your input proved to be of great value, and we have duly implemented the requisite adjustments to elevate the clarity of our data presentation.  Figure 2 – Line 337, Page 18  Figure 4 – Line 364 Page 21 |
| **REVIEWER #4** | |
|  |  |
| **Comments from the Reviewer 4** | **Comments of the authors** |
| The manuscript does not state why forecasting weekly dengue virus (DENV) incidence is essential for preventive and therapeutic purposes. DENV outbreaks in endemic countries, most of the time, follow seasonal patterns and just retrospectively inspecting epidemiologically data is enough to identify critical periods and consequently work on preparedness. Just looking figure 6, I don't find any benefit comparing predictions and real data. Unfortunately, the structure of this manuscript is not helpful understanding the usefulness of predicting weekly incidence. I would expected a reliable justification (e.g., identify hotspots to invest in infrastructure or human resources) but authors just mention an information gap. In the lack of sound rationale, I found very difficult to provide specific feedback to improve the manuscript. | We express our gratitude for your invaluable comment. In consideration of your suggestion, we have incorporated the following rationale into the text to elucidate the significance of weekly forecasting:  " Typically, dengue exhibits a seasonal pattern due to the association between mosquito breeding and the cyclical nature of rainfall. Nevertheless, climate change has the potential to induce phenological shifts in various species. Consequently, the anticipation of weekly dengue incidence assumes significant importance, particularly for the timely issuance of public warnings and notifications to public health authorities [14]. This is especially pertinent in countries with limited resources, such as Sri Lanka, where it is imperative to gain a comprehensive understanding of the dynamics of dengue transmission to devise more efficient control strategies. Therefore, comprehending the disease's propagation patterns within shorter time intervals, notably on a weekly basis, is crucial for the prevention and management of dengue in Sri Lanka.”  We have cited Iler, A. M., Caradonna, P. J., Forrest, J. R. K., & Post, E. (2021) to support this rationale in our revised manuscript (line 199). Thank you for your feedback. |
| 1. The introduction section should follow a conventional structure: disease burden, knowledge gap, how the gap was tackled previously, and the aims of this manuscript to tackle the gap innovately. Instead of that, irrelevant aspects are discussed (how virulence or new strains could be identified by using this model?), using non-conventional terms (ferocity?), and use interchangeably (but erroneously) basic epidemiolgical concepts (incidence and prevalence). For example, in DENV, how prevalence could help improving prevention and treatment? Are you referring to seroprevalence? It is unreasonable to measure DENV prevalence as this is not a chronic infection. Authors should clearly state how weekly DENV incidence prediction will impact prevention/treatment. | We extend our gratitude for your feedback pertaining to the organisation and content of the introduction section. We highly value your insights and the opportunity they present for enhancing the overall clarity and relevance of our manuscript.  In response to your concerns, we have undertaken a thorough revision of the introduction section to conform to a more standard structure. This entailed a deliberate focus on fundamental elements, including the disease burden, identification of existing knowledge gaps, previous efforts to address these gaps, and the innovative objectives outlined in our manuscript.  We also recognised that certain aspects discussed in the prior introduction may not have had direct relevance to the central focus of the paper. Notably, we refrained from delving into topics such as virulence or the emergence of new strains in the introduction, as these elements did not directly align with the core objectives of our study.  Furthermore, we took measures to ensure the correct and consistent use of basic epidemiological terminology. We acknowledge the misuse of terms like incidence and prevalence and have diligently made the necessary corrections.  In response to your inquiry concerning the utility of DENV prevalence, we value your observation, and we have provided a lucid explanation elucidating how the prediction of weekly DENV incidence profoundly impacts prevention and treatment strategies. This explanation emphasises the timeliness and effectiveness of early predictions in informing public health authorities and enabling the implementation of more efficient control measures.  Our commitment to enhancing the introduction section was rooted in the aim of aligning it more closely with the conventional structure while also elucidating the pivotal importance of our research within the context of prevention and treatment. Your feedback has been instrumental in effecting these improvements, resulting in a more robust and coherent introduction. |
| 2. Unless dictated by the editors, results and discussion should be separate sections. | We extend our sincere appreciation for the reviewer's valuable comment. We have diligently examined your suggestion to segregate the results and discussion sections, a practice commonly employed in many scholarly manuscripts.  After thorough deliberation, we have come to the conclusion that, given the specific context and content of our study, presenting both results and discussion within a single section yields a more coherent and succinct exposition of our findings and their interpretation. This integrated approach fosters a smoother flow of information and enhances the seamless integration of results with their corresponding discussions.  We recognise that diverse studies may benefit from varied structural arrangements, and in the case of our research, the integrated approach aligns well with the effective presentation of our research findings.  Your feedback is held in high regard, and we have duly considered it when determining the most suitable structural framework for our manuscript. |
|  |  |
| 3. Page 17, "Four dengue virus serotypes have been reported, marked as DENV 1 – 4. One of those serotypes predominated the outbreaks in specific years [20]. Since the severity of an epidemic tends to be associated with its serotype, mathematical modelling can be a reliable tool to make hypotheses about the emergence of new strains.": this is incorrect. Disease severity is explained by the ocurrence of secondary heterologous infections and inadequate medical interventions. Thus, the best way to predict severity is to conduct seroprevalence studies complemented by sentinel surveillance to identify different serotypes. I don't find any rationale in using this model to hypothesise new strains | We extend our profound gratitude for the comprehensive assessment and constructive feedback generously provided by the esteemed reviewers.  The reviewer aptly draws attention to the critical distinction between the severity of a disease and the severity of a disease outbreak, a differentiation we duly acknowledge. In our reference to the severity of an epidemic within the context of serotype dominance, we intend to encompass a multifaceted perspective, including considerations such as disease impact, transmissibility, economic and societal consequences, geographic reach, response capability, population susceptibility, and the availability of vaccination and treatment resources, among other factors (Thompson, R.N., Gilligan, C.A., and Cunniffe, N.J., 2020). It is this collective convergence of factors that contributes to the overall assessment of the severity and repercussions of an epidemic.  While we hold in high regard the reviewer's viewpoint on the value of seroprevalence studies and sentinel surveillance in assessing disease severity, our approach centres on mathematical modelling as a complementary tool. This modelling perspective is employed to formulate hypotheses about the emergence of new strains in the broader context of disease outbreak severity. Our approach aims to consider the far-reaching implications of new strains on epidemic severity, taking into account an array of factors that extend beyond individual disease severity.  We acknowledge the significance of various methodological approaches in comprehending and managing diseases and value the input provided by the reviewer. However, it is our contention that our emphasis on the utility of mathematical modelling, as elucidated in the provided reference, is well-aligned with the investigation of disease outbreak severity.  We remain open to further discussion and stand ready to address any additional concerns or queries raised by the reviewer. |
| 4. Policy implementation section: how forecasting weekly DENV incidence could complement or replace (partially or totally) epidemiologic surveillance? As shown in figure 6, I don't find any benefit simply inspecting retrospectively data compared with the modelling conducted by authors. Due to historical trends, endemic countries are well-informed of critical periods and simply introducing preventive actions before this periods could change morbidity and mortality. Unfortunately, politics and government priorities are out of the scope of this manuscript, authors should realise that mortality/morbidity is consequence of government passivity and not by gaps in information. | In the first instance, it is imperative to clarify that our intent was not to supplant epidemiologic surveillance with forecasting; rather, our primary objective was to augment and enhance it. To illustrate this, when our model anticipates the potential onset of an outbreak, it serves as an alert mechanism, prompting public health authorities to intensify their surveillance efforts, especially in regions flagged as potential hotspots by the model. The 'information gap' referred to in our manuscript stems from historical observations indicating that, during periods lacking predictive information, public health officials often allocated fewer resources to dengue control efforts. Our overarching goal is to bridge this gap by offering valuable insights that can bolster prevention and response measures.  We acknowledge the reviewer's viewpoint concerning government passivity and its potential contribution to morbidity and mortality. We also recognise that the complexities of policy implementation and government priorities extend far beyond the scope of our manuscript. However, we remain steadfast in our belief that enhanced forecasting can play a pivotal role in guiding more proactive strategies within the realm of public health. Ultimately, our emphasis lies in how predictive modelling can contribute to more informed decision-making, leading to the implementation of more effective prevention and control measures.  We greatly appreciate the reviewer's feedback and aim to convey the substantial benefits of forecasting as a complementary tool to epidemiologic surveillance, contributing to the advancement of public health strategies and objectives. |
