# Supplementary material for: Forecasting weekly dengue incidence in Sri Lanka: Modified Autoregressive Integrated Moving Average modeling approach
Source: PLoS One. 2024 Mar 8;19(3):e0299953. doi: 10.1371/journal.pone.0299953 (PMC10923413; doi:10.1371/journal.pone.0299953)
Supplement: Attachment — Submitted filename: Response to Reviewers.docx [file pone.0299953.s005.docx]

**Point by point response to editor and reviewers.**

Dear Editor and the Reviewers,

We extend our sincere gratitude to the editor for allowing us to undertake revisions and resubmit our manuscript. Our appreciation is also extended to the reviewers for their invaluable feedback, consisting of constructive comments and suggestions, which proved instrumental in enhancing the quality of our paper. Enclosed herewith is a comprehensive account detailing the manner in which we have addressed each comment in the revised version of the manuscript.

| **Comments from the Editor** | **Comments of the authors** |
| --- | --- |
| We note that the Academic Editor and one or more reviewers has recommended that you cite specific previously published works. As always, we recommend that you please review and evaluate the requested works to determine whether they are relevant and should be cited. It is not a requirement to cite these works.  We note that your previous response to comments by Reviewer 3 indicates you considered the reviewer's suggested citation to be relevant; please clarify this if needed in light of the further comments by Reviewer 3 appended below. We appreciate your attention to this request. | We appreciate your feedback. The paper titled 'Modeling and Forecasting the Weekly Incidence of Dengue in Colombo District of Sri Lanka' was presented and incorporated into the proceedings of the conference on Advancements in Science and Humanities at SLIIT, Malabe, Sri Lanka (pages 220-225) in November 2022. In alignment with your suggestion, the authors of the present manuscript concur that citing this work within the current document is unnecessary. The reference is as follows,  Arachchi, K. A. N. L. K., & Peiris, T. S. G. (2022). Modeling and Forecasting the Weekly Incidence of Dengue in Colombo District of Sri Lanka. In The Proceedings of the Conference on Advancements in Science and Humanities (pp. 220-225). SLIIT, Malabe, Sri Lanka.  Please find our response to Reviewer 3 outlined below. |
| **REVIEWER #3** | |
| **Comments from the Reviewer 3** | **Comments of the authors** |
| In the response to reviewers' letter the authors state, "Furthermore, we have made a conscientious effort to reference pertinent research, including the work by Lynch et al. (2021), in order to underscore the critical importance of taking into account the geographic level in the domain of virus forecasting." However, this reference does not appear in the newly added 'Limitations of the Study' section of the paper which was meant to address the concern identified in the reference and it does not appear elsewhere in the paper. The paper needs to be revised to be consistent with the modifications the authors have described in the response to reviewers' letter. | We appreciate your diligence in bringing to our attention the oversight on our part. Regrettably, the reference was inadvertently omitted in the last manuscript.  The reference has now been incorporated into the revised version; kindly refer page 23 of the Track version.  The newly included content is articulated as follows: “Moreover, within the scope of this research, projections were executed at a consistent geographical level, specifically confined to the Colombo district. This approach contrasts with the work undertaken by Lynch et al. (2021), where time series models were formulated for forecasting dengue incidents, transcending country-level, health district, and state-level considerations. It is noteworthy that, unlike COVID, dengue is not equally prevalent across all districts in Sri Lanka; its primary occurrence is concentrated in the Colombo and Gampaha districts. To facilitate the development of a unified model applicable to both district a dummy variable can be considered along with weekly dengue incidents for Gampaha district.”  The primary objective of this study was to develop a model specifically designed for the Colombo district, given its consistently highest percentage of dengue incidences over the years.. An additional remark has been incorporated in the concluding lines of the "Limitation of this study" section to elucidate this aspect. |
| **REVIEWER #4** | |
| **Comments from the Reviewer 4** | **Comments of the authors** |
| I don't think my first and third comments were resolved.  The introduction still discusses irrelevant aspects and inaccurately attempts to justify the usefulness of forecasting dengue incidence (prevalence is still used).  Regarding my third comment, I'm afraid I disagree that severity, which is an eminently clinical definition, should be interpreted in terms of disease impact or consequences. Those aspects are the burden of disease, and it is not justifiable to use disease burden and disease severity interchangeably. | We appreciate your comment, and it has been duly noted. To address this concern, we have included an additional statement on forecasting, which is explained in paragraph 2 on page 4.  Furthermore, in alignment with your recommendations, we have eliminated the term "prevalence" where it originally occurred, by exclusively replacing the term with "incidence."  Additionally, the last line of paragraph 2 in the Introduction on page 3 has been removed in accordance with your suggestions. |
| 1. The introduction section should follow a conventional structure: disease burden, knowledge gap, how the gap was tackled previously, and the aims of this manuscript to tackle the gap innovatively. Instead of that, irrelevant aspects are discussed (how virulence or new strains could be identified by using this model?), using non-conventional terms (ferocity?), and use interchangeably (but erroneously) basic epidemiological concepts (incidence and prevalence). For example, in DENV, how prevalence could help improving prevention and treatment? Are you referring to seroprevalence? It is unreasonable to measure DENV prevalence as this is not a chronic infection. Authors should clearly state how weekly DENV incidence prediction will impact prevention/treatment.    ) | We express our sincere appreciation for your valuable feedback on both the organisation and content of the introduction section. Your insights are highly valued, presenting an opportunity to enhance the overall clarity and relevance of our manuscript.  In response to your thoughtful concerns, we conducted a comprehensive revision of the introduction section to adhere to a more standardised structure. This involved a deliberate focus on fundamental elements, including the disease burden, identification of existing knowledge gaps, previous efforts to address these gaps, and the innovative objectives outlined in our manuscript.  Recognising that certain aspects discussed in the previous introduction may not directly align with the central focus of the paper, we refrained from delving into topics such as virulence or the emergence of new strains. It is essential to clarify that our model does not identify virulence or new strains directly, but rather, mathematical modelling like ours could be used to hypothesise the emergence of new strains. As aptly stated, "Given that the severity of an epidemic often correlates with the prevailing serotype, mathematical modelling can serve as a valuable instrument for formulating hypotheses regarding the emergence of new strains."  We appreciate your correction regarding the use of "ferocity," and was replaced with the more appropriate term, "virulence." in the previous draft.  Regarding the distinction between incidence and prevalence, we acknowledge the importance of accurate usage. We have corrected our terminology, understanding that Dengue, being an acute condition without chronic clinical implications, is more appropriately characterised by incidence rather than prevalence. We also appreciate your clarification on seroprevalence and its distinction from incidence.  Furthermore, we have taken measures to ensure the correct and consistent use of basic epidemiological terminology. Your guidance on the misuse of terms like incidence and prevalence has been duly acknowledged and rectified.  Prevalence encompasses all cases, including both pre-existing and new instances, that exist at a given time, whereas incidence specifically pertains to new cases. Given that Dengue does not lead to chronic clinical conditions and is confined to acute conditions, the term "incidence" is more apt in this context. It is important to note that seroprevalence, on the other hand, refers to the proportion of individuals in a population with Dengue antibodies, specifically IgG in past infections and IgM in current infections. Detecting these antibodies involves a blood test, making it a challenging task to determine the seroprevalence of an entire population, except through estimation based on a sample (<https://doi.org/10.1016/j.jiph.2023.08.020>)  In response to your inquiry on the utility of DENV prevalence, we have provided a clear explanation elucidating how the prediction of weekly DENV incidence significantly impacts prevention and treatment strategies. This clarification underscores the timely and effective nature of early predictions in informing public health authorities and facilitating the implementation of more efficient control measures. |
| 3. Page 17, "Four dengue virus serotypes have been reported, marked as DENV 1 – 4. One of those serotypes predominated the outbreaks in specific years [20]. Since the severity of an epidemic tends to be associated with its serotype, mathematical modelling can be a reliable tool to make hypotheses about the emergence of new strains.": this is incorrect. Disease severity is explained by the occurrence of secondary heterologous infections and inadequate medical interventions. Thus, the best way to predict severity is to conduct seroprevalence studies complemented by sentinel surveillance to identify different serotypes. I don't find any rationale in using this model to hypothesise new strains. | We express our sincere appreciation for the thorough evaluation and valuable feedback provided by the esteemed reviewers.  The reviewer accurately highlights the crucial distinction between the severity of a disease and the severity of a disease outbreak, a differentiation we duly recognise. In referring to the severity of an epidemic in the context of serotype dominance, our intention is to encompass a comprehensive perspective, considering factors such as disease impact, transmissibility, economic and societal consequences, geographic reach, response capability, population susceptibility, and availability of vaccination and treatment resources, among other considerations (Thompson, R.N., Gilligan, C.A., and Cunniffe, N.J., 2020). It is the amalgamation of these factors that contributes to the overall assessment of the severity and repercussions of an epidemic.  While we hold the reviewer's perspective on the value of seroprevalence studies and sentinel surveillance in high regard, our approach centres on mathematical modelling as a complementary tool. This modelling perspective is employed to formulate hypotheses regarding the emergence of new strains in the broader context of disease outbreak severity. Our argument emphasises that mathematical modelling can be instrumental in hypothesising the emergence of new strains with different virulence. In other words, if disease occurrence exceeds the model's explanatory capacity, it may be attributable to the emergence of new strains with distinct virulence.  We acknowledge the significance of various methodological approaches in comprehending and managing diseases and appreciate the input provided by the reviewer. However, we contend that our emphasis on the utility of mathematical modelling, as elucidated in the provided reference, aligns well with the investigation of disease outbreak severity.  We remain open to further discussion and are prepared to address any additional concerns or queries raised by the reviewer. |
